# Erratum: Bujnovsky, D., et al. Physical Fitness Characteristics of High-level Youth Football Players: Influence of Playing Position. *Sports* 2019, *7*, 46

**DOI:** 10.3390/sports7120250

**Published:** 2019-12-11

**Authors:** David Bujnovsky, Tomas Maly, Kevin R. Ford, Dai Sugimoto, Egon Kunzmann, Mikulas Hank, Frantisek Zahalka

**Affiliations:** 1 Faculty of Physical Education and Sport, Charles University, 162 52 Prague, Czech Republic; bujnovsky@ftvs.cuni.cz (D.B.); egonkunzmann@gmail.com (E.K.); hank@ftvs.cuni.cz (M.H.); zahalka@ftvs.cuni.cz (F.Z.); 2 Department of Physical Therapy, High Point University, High Point, NC 27268, USA; kford@highpoint.edu; 3 The Micheli Center for Sports Injury Prevention, Waltham, MA 02453, USA; dai.sugimoto@childrens.harvard.edu; 4 Department of Orthopaedic Surgery, Harvard Medical School, Boston, MA 02115, USA; 5 Division of Sports Medicine, Department of Orthpaedics, Boston Children’s Hospital, Boston, MA 02115, USA

The authors wish to make the following correction to this paper [1]: The author name “David Bujnovky” should read “David Bujnovsky”.

The authors and the Editorial Office would like to apologize for any inconvenience caused to the readers by these changes. The change does not affect the scientific results. The manuscript will be updated and the original will remain online on the article webpage.
